# The Diagnostic Accuracy of Serum and Urine Human Epididymis Protein 4 (HE4) in Ovarian Cancer in 15,394 Subjects: An Updated Meta-Analysis

**DOI:** 10.7759/cureus.30457

**Published:** 2022-10-19

**Authors:** Neelam Nalini, Amit Kumar, Saumya Sharma, Bijeta Singh, Aditya V Singh, Jay Prakash, Shreshtha Singh

**Affiliations:** 1 Obstetrics and Gynaecology, Rajendra Institute of Medical Sciences, Ranchi, IND; 2 Laboratory Medicine, Rajendra institute of Medical Sciences, Ranchi, IND; 3 Obstetrics and Gynaecology, Medinirai Medical College, Ranchi, IND; 4 Medicine, Laxmi Chandravanshi Medical College, Ranchi, IND; 5 Critical Care Medicine, Rajendra Institute of Medical Sciences, Ranchi, IND; 6 Anaesthesia, John Hunter Hospital, New South Wales, AUS

**Keywords:** carbohydrate antigen 125, transvaginal sonography, ovarian cancer, he4, biomarker

## Abstract

Background

We aim to determine the diagnostic accuracy of both serum and urinary human epididymis protein 4 (HE4) in the diagnosis of ovarian cancer.

Methods

Electronic databases and search engines such as PubMed, Cochrane Library, and Google Scholar were searched systematically by two independent reviewers to retrieve articles published from inception to June 11, 2022. The diagnostic accuracy of serum and urinary HE4 was computed using the random-effects model in terms of pooled sensitivity, pooled specificity, and diagnostic odds ratio (DOR) with 95% confidence interval (CI). To explain any source of possible heterogeneity, meta-regression and subgroup analyses were performed. Risk of bias assessment was conducted using the Quality Assessment of Diagnostic Accuracy Studies 2 (QUADAS-2) tools recommended by the Cochrane Library.

Result and conclusion

This meta-analysis included a total of 38 studies of serum HE4 involving 14,745 subjects and five studies for urinary HE4 involving 649 subjects. We observed acceptable pooled sensitivity, specificity, summary receiver operating characteristics (SROC), and diagnostic odds ratio (DOR) at 0.79 (95% CI: 0.75-0.82), 0.92 (95% CI: 0.87-0.95), 0.88 (95% CI: 0.85-0.91), and 43 (95% CI: 25-72), respectively, for serum HE4 for discriminating ovarian cancer. For urine HE4, the pooled sensitivity, specificity, SROC, and DOR were 0.80 (95% CI: 0.64-0.90), 0.93 (95% CI: 0.83-0.98), 0.94 (95% CI: 0.91-0.95), and 55 (95% CI: 15-198), respectively. Therefore, HE4 is a promising biomarker with a high degree of specificity and acceptable sensitivity for the diagnosis of ovarian cancer.

Registration number

This meta-analysis was performed after the registration of the protocol in the PROSPERO database with registration number CRD42022324947.

## Introduction and background

Ovarian cancer is associated with very high mortality because of late diagnosis and the absence of any standard method of screening such as a Papanicolaou (Pap) smear for cervical cancer. It is the leading cause of mortality related to gynecological malignancy [1]. For average-risk women, routine screening for ovarian cancer is not recommended currently. The Food and Drug Administration (FDA) in September 2016 [2] issued a recommendation against any routine screening test for ovarian cancer in average-risk women. The reason behind this was the inaccurate results of available biomarkers. Ovarian cancer is the eighth most common cause of death globally [3]. The two most common investigations that are used to differentiate between benign and malignant pelvic or ovarian masses are serum carbohydrate antigen 125 (CA-125) and transvaginal sonography. However, both of these methods have their own limitations [4-6].

There is a significant decrease in death due to cervical cancer worldwide. This became possible due to the widespread availability and standard interpretation of good screening tests, Pap smear. Therefore, today’s utmost need is to do more research on ovarian cancer (using standard methodology) and do a meta-analysis and systematic review to reach a robust conclusion and formulate a standard screening test with discriminatory power to differentiate between benign and malignant ovarian tumors. This HE4 was approved by the FDA [7] in 2008 for screening and diagnosing ovarian cancer. The high specificity of HE4 in diagnosing ovarian cancer drew our attention to do an updated meta-analysis. Different systematic reviews and meta-analyses of HE4 to know its diagnostic accuracy have also been performed before [4,8-16]. However, various methodological shortcomings were also there. Most recent and recommended meta-analysis methods were not used in the previous meta-analysis [17].

After the publication of these meta-analyses, further studies have appeared in the literature, and there is a need to update the meta-analysis for precise evidence. Therefore, our team did an updated meta-analysis about the diagnostic accuracy of both serum and urine HE4 in ovarian cancer.

## Review

Methods

The protocol of this meta-analysis was registered in the PROSPERO database with registration number CRD42022324947. We followed the Preferred Reporting Items for Systematic Reviews and Meta-Analyses (PRISMA) guidelines [18]. The research questions of our current meta-analysis were formulated according to the PICO tool, which stands for patients/populations/problems (cases of ovarian cancer), index test (in the meta-analysis, HE4 levels in serum and urine of subjects are used as index test; the cutoff value of HE4 was different in various studies depending upon the method used for measurement), comparator (gold standard was a biopsy and histopathological examination (HPE)), and outcome (the patient-oriented outcome was determined by pooled sensitivity and specificity). The design of the present study is an updated meta-analysis.

Selection of Studies and Search Strategy

Two authors independently searched Google Scholar, PubMed, and Cochrane Library to obtain all the relevant published articles published from inception to June 11, 2022. The keywords and filters searched included the following: ovarian cancer OR pelvic masses OR ovarian malignancy AND urine HE4 AND serum HE4. Efforts had been made to search for references of matched concerning articles to avoid missing any study related to HE4. The full text of all the articles was reviewed for evaluation of inclusion and exclusion criteria. Quality assessment was done for the final selection of articles. We included the studies if they met the inclusion and exclusion criteria.

Inclusion criteria: Studies published in the English language, only original articles, only full-text articles, studies conducted on both premenopausal (>18 years) and postmenopausal women, and articles of sufficient information to calculate sensitivity and specificity were included.

Exclusion criteria: We excluded case reports, case series, preprint studies, conference proceedings, studies with insufficient data, duplicate studies, letters to the editor, and studies about the role of other tumor markers (other than HE4) in ovarian malignancy.

Quality Assessment of Studies

Quality assessment was conducted by two independent reviewers using the Quality Assessment of Diagnostic Accuracy Studies 2 (QUADAS-2) tool [19]. A third reviewer was consulted to reach a conclusion in case of discrepancy. We followed QUADAS-2 to evaluate the risk of bias and the accuracy of diagnostic studies. It consists of four key domains: selection of patient, index test, reference standard, and flow and timing.

Extraction of Data

Independent extraction of data was done by two independent researchers. The following data were extracted from the eligible studies: author’s name, country, year of study, sample size (cases of ovarian cancer), controls, premenopausal/postmenopausal status, mean age, method of assessment, cutoff value, type of hospital setup, gold standard, type of ovarian cancer, study design, family history, and ethnicity (Tables 1, 2). Any disagreements between the two researchers were resolved by consulting a third researcher.

**Table 1:** Characteristics of studies on serum HE4 Study design: 1, cross-sectional study; 2, prospective observational study; 3, analytical comparative study; 4, case-control study Method of assessment: 1, ELISA; 2, CMIA; 3, ECLIA HE4, human epididymis protein 4; TP, true positive; FN, false negative; TN, true negative; FP, false positive; ELISA, enzyme-linked immunosorbent assay; CMIA, chemiluminescent microparticle immunoassay; ECLIA, electrochemiluminescence immunoassay [5,20-56]

Author name	Country	Year	Sample size	Ovarian cancer	Control	Sensitivity	Specificity	TP	FN	TN	FP	Mean age (years)	Assessment method	Cutoff (pmol/L)	Hospital setup	Gold standard	Ovarian cancer type	Study design	Ethnicity	Premenopausal status	Postmenopausal status
Pitta et al. [20]	Brazil	2013	326	60	266	0.583	0.845	35	25	225	41	47.7	1	Premenopausal: 41.6, postmenopausal: 96.6	Tertiary center	Tissue biopsy	Serous epithelial	1	Brazilian	52.14	47.85
Dewan et al. [21]	India	2019	135	67	68	0.836	1	56	11	68	0	45.8	2	70	Tertiary center	Tissue biopsy	Serous epithelial	2	Asian		
Chen et al. [22]	China	2014	232	60	172	0.883	0.971	53	7	167	5	49.5	3	87.6		Tissue biopsy		2			
Terzic et al. [23]	Serbia	2013	358	52	306	0.7018	0.8891	36	16	272	34		1	150	Tertiary center	Tissue biopsy	Serous epithelial	2			
Shen et al. [24]	China	2017	684	202	482	0.89	0.8004	180	22	386	96		2	Premenopausal: 70, postmenopausal: 140		Tissue biopsy		2			
Bandiera et al. [25]	Italy	2011	419	279	140	0.846	0.942	236	43	132	8										
Lawicki et al. [26]	Poland	2013	180	100	80	0.7	0.94	70	30	75	5		2	75.75	Tertiary center	Tissue biopsy	Serous epithelial				
Abdalla et al. [27]	Poland	2018	302	50	248	0.7	0.925	35	15	229	19	48.7	3	Premenopausal: 70, postmenopausal: 140		Tissue biopsy				62.26	37.74
Abdel-Azeez et al. [28]	Egypt	2010	90	41	49	0.829	0.875	34	7	43	6		1	72	Tertiary center	Tissue biopsy	Serous epithelial	2			
Ahmed et al. [29]	Egypt	2019	140	62	78	0.839	0.705	52	10	55	23		1	150	Tertiary center	Tissue biopsy		2		50	50
Aslan et al. [30]	Turkey	2020	84	19	65	0.842	0.984	16	3	64	1		1	150	Tertiary center	Tissue biopsy	Serous epithelial	2			
Oranratanaphan et al. [31]	Thailand	2018	281	56	225	0.741	0.865	41	15	195	30	44	3	70	Tertiary center	Tissue biopsy		2		75.8	24.2
Priyanka et al. [32]	India	2017	35	16	19	0.63	0.88	10	6	17	2		2	Premenopausal: 70, postmenopausal: 140	Tertiary center	Tissue biopsy	Serious epithelial	2	Asian	62.90	37.1
Wilailak et al. [33]	Asian countries (multicentric)	2014	414	65	349	0.837	0.949	54	11	331	18	41.2	1			Tissue biopsy		2			
Wei et al. [34]	China	2016	158	64	94	0.729	0.95	47	17	89	5	55	2	140		Tissue biopsy	Serous cystadenocarcinoma				
Yanaranop et al. [35]	Thailand	2016	260	74	186	0.662	0.86	49	25	160	26	48.2		Premenopausal: 70, postmenopausal 140	Tertiary center	Tissue biopsy	Serous epithelial	1	Asian	56.90	43.1
Kaijser et al. [36]	Belgium	2014	360	144	216	0.74	0.85	107	37	184	32		1	114	Tertiary center	Tissue biopsy		1			
Goff et al. [37]	USA	2017	218	66	152	0.58	0.94	38	28	143	9	60	2	140	Tertiary center	Tissue biopsy			Mixed	16.7	83.3
Park et al. [38]	Korea	2011	2505	66	2439	0.909	0.941	60	6	2295	144	52.1	2	33.2	Tertiary center	Tissue biopsy		2			
Moore et al. [5]	USA	2008	233	67	166	0.729	0.95	49	18	158	8	49	1	70	Tertiary center	Tissue biopsy	Serous epithelial	1			
Nolen et al. [39]	USA	2010	405	264	141	0.705	0.85	186	78	120	21	62	1	38.5	Tertiary center	Tissue biopsy					
Jacob et al. [40]	Switzerland	2011	160	56	104	0.789	0.859	44	12	89	15		1	70		Tissue biopsy	Serous epithelial	2		52.50	47.5
Anastasi et al. [41]	Italy	2010	267	32	235	0.969		31	1	0	235		1	150		Tissue biopsy					
Andersen et al. [42]	USA	2010	211	74	137	0.77	0.949	57	17	130	7		1	Upper 95th percentile of the benign group				4			
Van Gorp et al. [43]	Belgium	2011	389	161	228	0.739	0.851	119	42	194	34		1	72.2	Tertiary center	Tissue biopsy	Serous epithelial			47.3	52.7
Chang et al. [44]	China	2011	491	52	439	0.82	0.98	43	9	430	9		1	150	Tertiary center	Tissue biopsy	Serous epithelial	3			
Holcomb et al. [45]	USA	2011	229	18	211	0.889	0.918	16	2	194	17		1	70				3		100	
Montagnana et al. [46]	Italy	2011	104	55	49	0.764	0.939	42	13	46	3	51.2	1	74.2	Tertiary center	Tissue biopsy	Serous epithelial			49.03	50.96
Dong et al. [47]	China	2008	277	30	247	0.7	1	21	9	247	0		1	86							
Mitre et al. [48]	Albania	2020	265	53	212	0.873	0.972	46	7	206	6		1	150							
Hamed et al. [49]	Egypt	2013	70	30	40	0.9	0.95	27	3	38	2	50.7	2	150	Tertiary center	Tissue biopsy	Serous epithelial			25	75
Anton et al. [50]	Brazil	2012	120	37	83	0.796	0.667	29	8	55	28	54.7	1	70	Tertiary center	Tissue biopsy		2		39.16	60.83
Ortiz-Muñoz et al. [51]	Spain	2014	218	32	186	0.862	0.874	28	4	163	23		3	140							
Molina et al. [52]	Spain	2011	527	111	416	0.793	0.989	88	23	411	5		2	150							
Karlsen et al. [53]	Denmark	2012	1218	252	966	0.944	0.632	238	14	611	355		2	150	Tertiary center	Tissue biopsy	Serous epithelial	2			
Gentry-Maharaj et al. [54]	United Kingdom	2020	1590	78	1512	0.538	0.9	42	36	1361	151	62				Tissue biopsy	Mixed	2		100	
Ruggeri et al. [55]	Italy	2011	259	96	163	0.823	0.982	79	17	160	3		2	87.4	Tertiary center	Tissue biopsy	Serous epithelial	2		45.2	54.8
Lenhard et al. [56]	Germany	2011	535	125	410	0.674	0.95	84	41	390	21	62.9	1							50.6	49.4
				3166	11579																

**Table 2:** Characteristics of studies on urine HE4 HE4, human epididymis protein 4; ELISA, enzyme-linked immunosorbent assay [57-61]

Author name	Country	Year	Sample size	Ovarian cancer	Control	Sensitivity	Specificity	Assessment method	Cutoff	Hospital setup	Gold standard	Ovarian cancer type	Study design	Ethnicity
Liao et al. [57]	USA	2015	356	169	187	52.2	95	ELISA	Urine HE4/creatinine: 3.5	Tertiary center	Tissue biopsy		Prospective cohort study	Caucasian
Fan et al. [58]	China	2017	67	31	36	83.9	100	Electrochemiluminescence immunoassay	14,116 pmol/L	Tertiary center	Tissue biopsy	Mixed	Case control	Asian
Hellstrom et al. [59]	USA	2010	109	53	56	88.6	94.40	ELISA	Not reported	Tertiary center	Tissue biopsy	Serous epithelial	Prospective cohort study	Caucasian
Macuks et al. [60]	Latvia	2012	78	23	55	78.30	75	ELISA	13,000 pmol/L	Tertiary center	Tissue biopsy		Case-control study	Caucasian
Wang et al. [61]	USA	2011	39	19	20	89.50	90	Microchip ELISA and cellphone/charge-coupled device-based calorimetric measurements	43.81 pmol/L	Tertiary center	Tissue biopsy		Prospective cohort study	Caucasian

Statistical Analysis

In the meta-analysis, we followed the standard method recommended for the meta-analysis of diagnostic tests. In this meta-analysis, we calculated the pooled sensitivity and pooled specificity with 95% confidence interval using a random-effects model. The diagnostic odds ratio (DOR), which is a measure of the effectiveness of any diagnostic test, is calculated according to the following formula: DOR=(true positive (TP)/false negative (FN))/(false positive (FP)/true negative (TN)). We computed positive and negative likelihood ratios as well (LR+ and LR-). The discriminatory accuracy of serum and urine HE4 was determined by constructing a summary receiver operating characteristics (SROC) curve. The heterogeneity among the different study groups was assessed using the LR I^2 ^index and chi-square test. When its value is >50%, it indicates the presence of heterogeneity. To explain any possible source that can explain heterogeneity, meta-regression and subgroup analyses were performed. The following moderators/variables were considered for the meta-regression analysis: mean age, cutoff value of the HE4 test, method of assessment, and ethnicity. Publication bias was assessed using Deek’s funnel plot. The analysis was done using Stata version 13 (StataCorp LLC, College Station, TX, USA).

Search Results for Serum HE4

**Figure 1:**
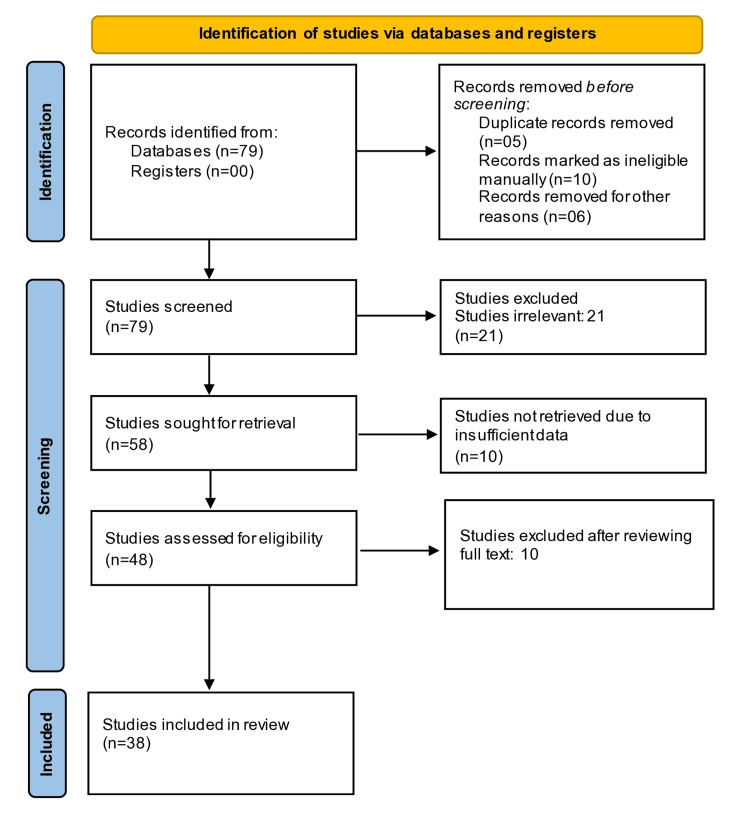
PRISMA for serum HE4 study selection and inclusion PRISMA, Preferred Reporting Items for Systematic Reviews and Meta-Analyses; HE4, human epididymis protein 4

A total of 79 studies were identified by electronic database and search engines, of which 18 studies were taken from the previous version of the review and eight studies from citation searching. Twenty-one studies were excluded, out of which 10 studies were marked ineligible manually and the rest were excluded by screening title and abstract. The full texts of 58 studies were reviewed, out of which 10 studies were removed because of insufficient data, leaving 48 studies for analysis. Further 10 studies were removed after reviewing the full text, leaving 38 studies for final analysis. Figure 1 provides the PRISMA chart of the literature search process.

Characteristics of Studies on Serum HE4

Details of the included studies on serum HE4 are provided in Table 1 [5,20-56]. These 38 studies analyzed serum HE4 (analysis of urine HE4 is shown in Table 2). Out of the 38 studies, five were cross-sectional, 16 were prospective observational, two were analytic comparative, and two were case-control. The number of patients included in different studies ranged from 16 to 279. The mean age of patients in the included studies ranged from 41.2 to 62.9 years. Out of the 38 studies, in eight studies, the selection process of control populations was not clearly defined (e.g., the association with other medical disorders, especially renal diseases and/or gynecological disorder, ultrasonographic findings to exclude any asymptomatic ovarian cyst/ovarian mass/any adnexal mass, and the age group of patients were not mentioned).

Search Results for Urine HE4

**Figure 2:**
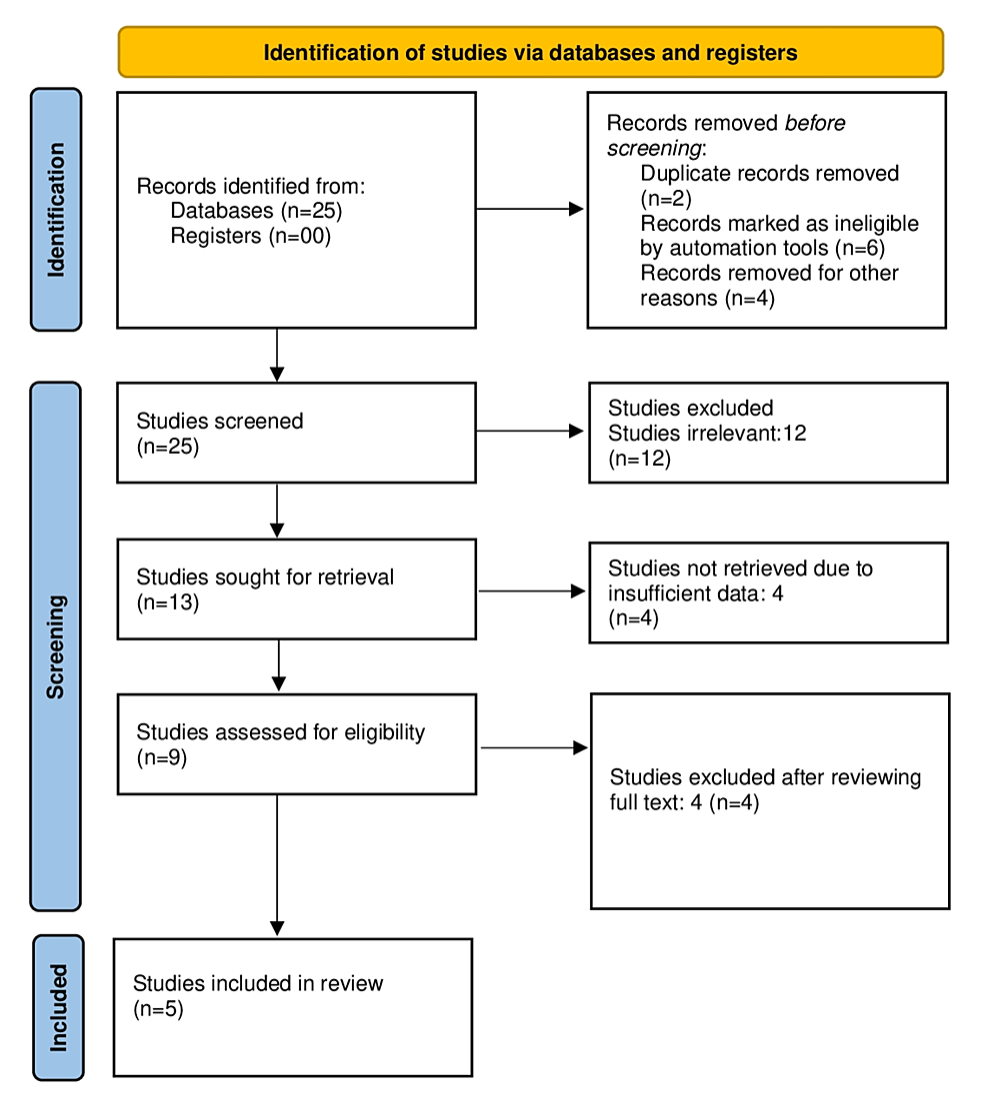
PRISMA for urine HE4 study selection and inclusion PRISMA, Preferred Reporting Items for Systematic Reviews and Meta-Analyses; HE4, human epididymis protein 4

A total of 25 studies were identified by electronic database and search engines, of which 16 studies were taken from the previous version of the review and nine studies from citation searching. Twelve studies were excluded, out of which 6 studies were marked ineligible manually and the rest were excluded by screening title and abstract. Thirteen studies were left for full-text review and data extraction. Finally, four studies were removed because of insufficient data, leaving nine studies for analysis. Further four studies were removed after reviewing the full text, leaving five studies for final analysis. Figure 2 provides the PRISMA chart of the literature search process.

Characteristics of Studies on Urine HE4

Details of the included studies on urine HE4 are provided in Table 2 [57-61]. The total number of participants involved in these five studies was 649. Of the five studies, two were case-control and three were prospective cohort studies. The number of patients included in the different studies ranged from 19 to 169. The mean age of patients in the included studies was not mentioned. In all five studies, the selection process of control populations was clearly defined, which included a healthy population and patients with a pelvic mass and other gynecological disorders.

Quality Assessment of the Included Studies

**Figure 3:**
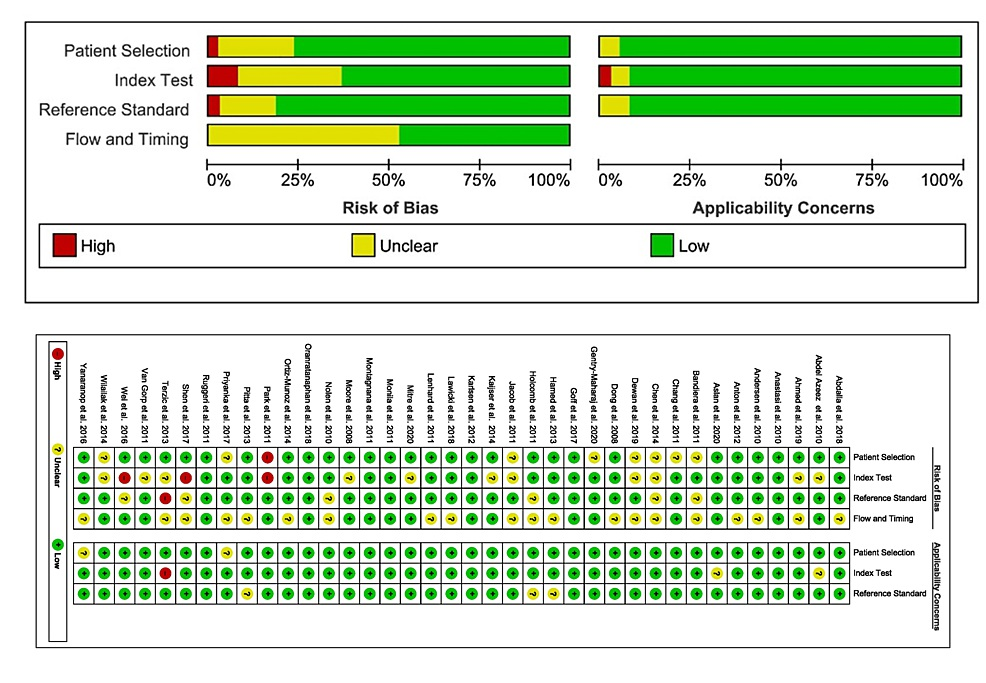
QUADAS-2 for quality assessment of serum HE4 QUADAS-2, Quality Assessment of Diagnostic Accuracy Studies 2; HE4, human epididymis protein 4 Yanaranop et al. [35], Wilailak et al. [33], Wei et al. [34], Terzic et al. [23], Shen et al. [24], Ruggeri et al. [55], Priyanka et al. [32], Pitta et al. [20], Park et al. [38], Ortiz-Muñoz et al. [51], Oranratanaphan et al. [31], Nolen et al. [39], Moore et al. [5], Montagnana et al. [46], Monila et al. [52], Mitre et al. [48], Gentry-Maharaj et al. [54], Lenhard et al. [56], Lawicki et al. [26], Karlsen et al. [53], Kaijser et al. [36], Jacob et al. [40], Holcomb et al. [45], Hamed et al. [49], Van Gorp et al. [43], Goff et al. [37], Dong et al. [47], Dewan et al. [21], Chen et al. [22], Chang et al. [44], Bandiera et al. [25], Aslan et al. [30], Anton et al. [50], Andersen et al. [42], Anastasi et al. [41], Ahmed et al. [29], Abdel-Azeez et al. [28], Abdalla et al. [27]

**Figure 4:**
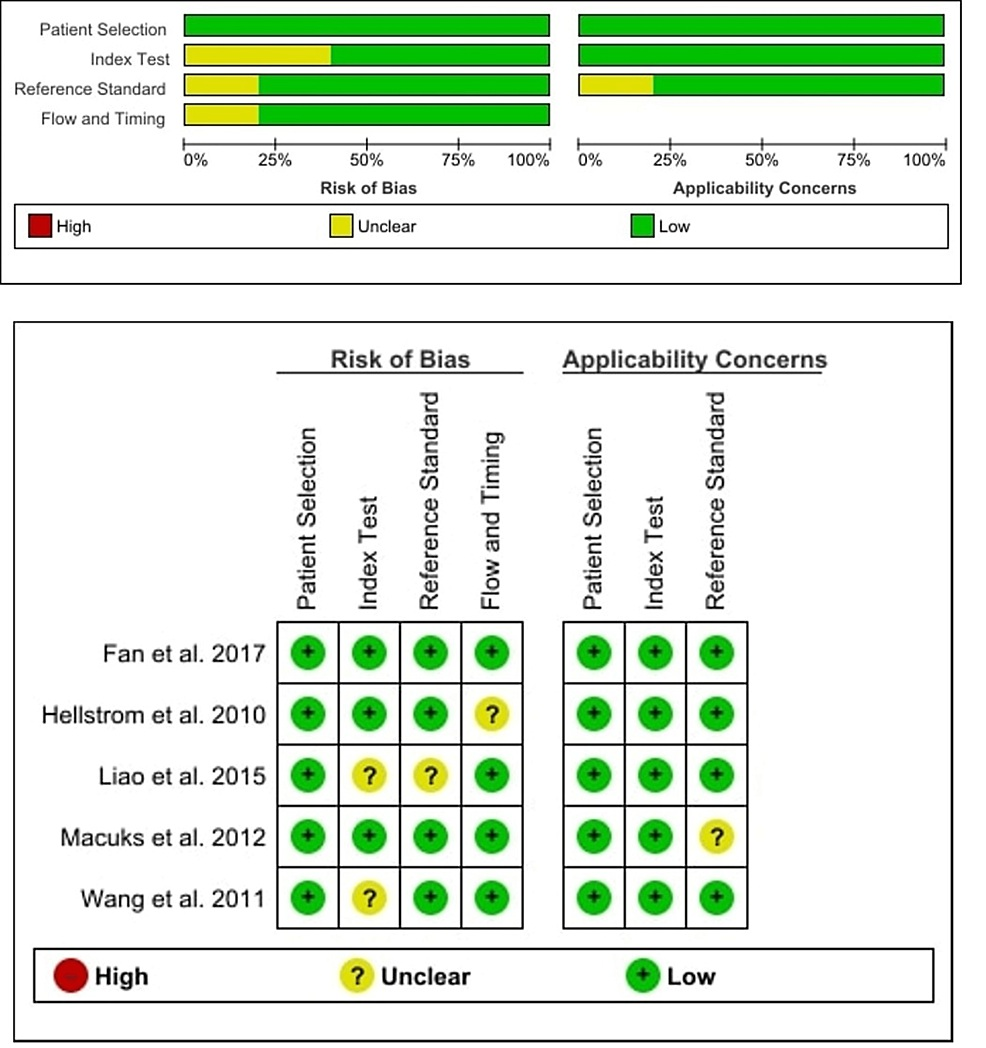
QUADAS-2 for quality assessment of urine HE4 QUADAS-2, Quality Assessment of Diagnostic Accuracy Studies 2; HE4, human epididymis protein 4 Liao et al. [57], Fan et al. [58], Hellstrom et al. [59], Macuks et al. [60], Wang et al. [61]

Figure 3 shows the detailed result of the QUADAS-2 assessment of serum HE4, and Figure 4 shows the detailed result of the QUADAS-2 assessment of urine HE4. These QUADAS-2 results demonstrated that the qualities of the included studies were adequate.

Results

*Result of Serum HE4* *Meta-Analysis*

**Figure 5:**
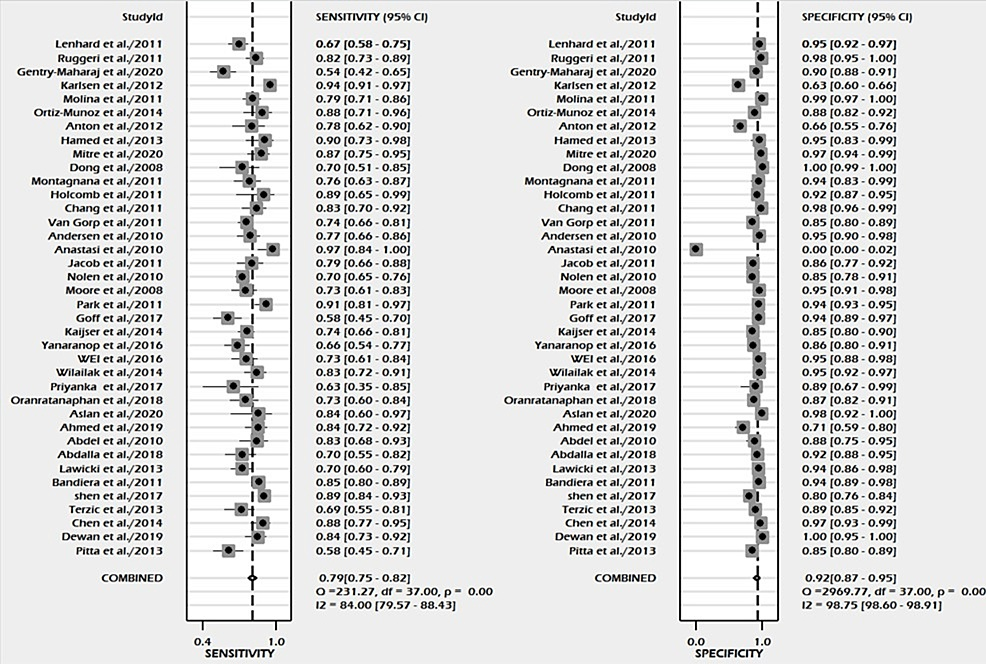
Forest plot of pooled sensitivity and specificity of serum HE4 in the diagnosis of ovarian cancer HE4, human epididymis protein 4; CI, confidence interval [5,20-56]

**Figure 6:**
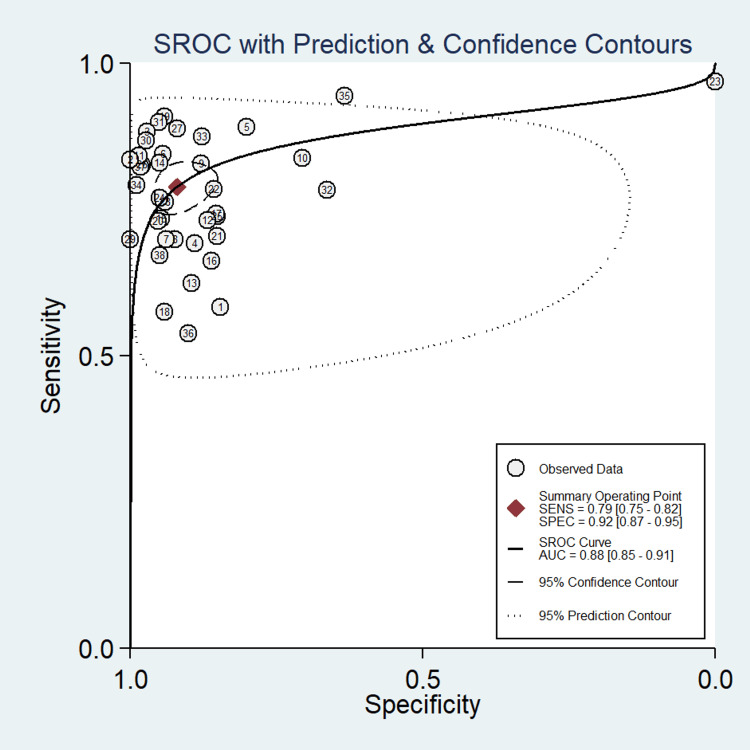
SROC curve with prediction and confidence contours showing the discriminatory power of serum HE4 for diagnosing ovarian cancer SROC, summary receiver operating characteristics; HE4, human epididymis protein 4; AUC, area under the curve

**Figure 7:**
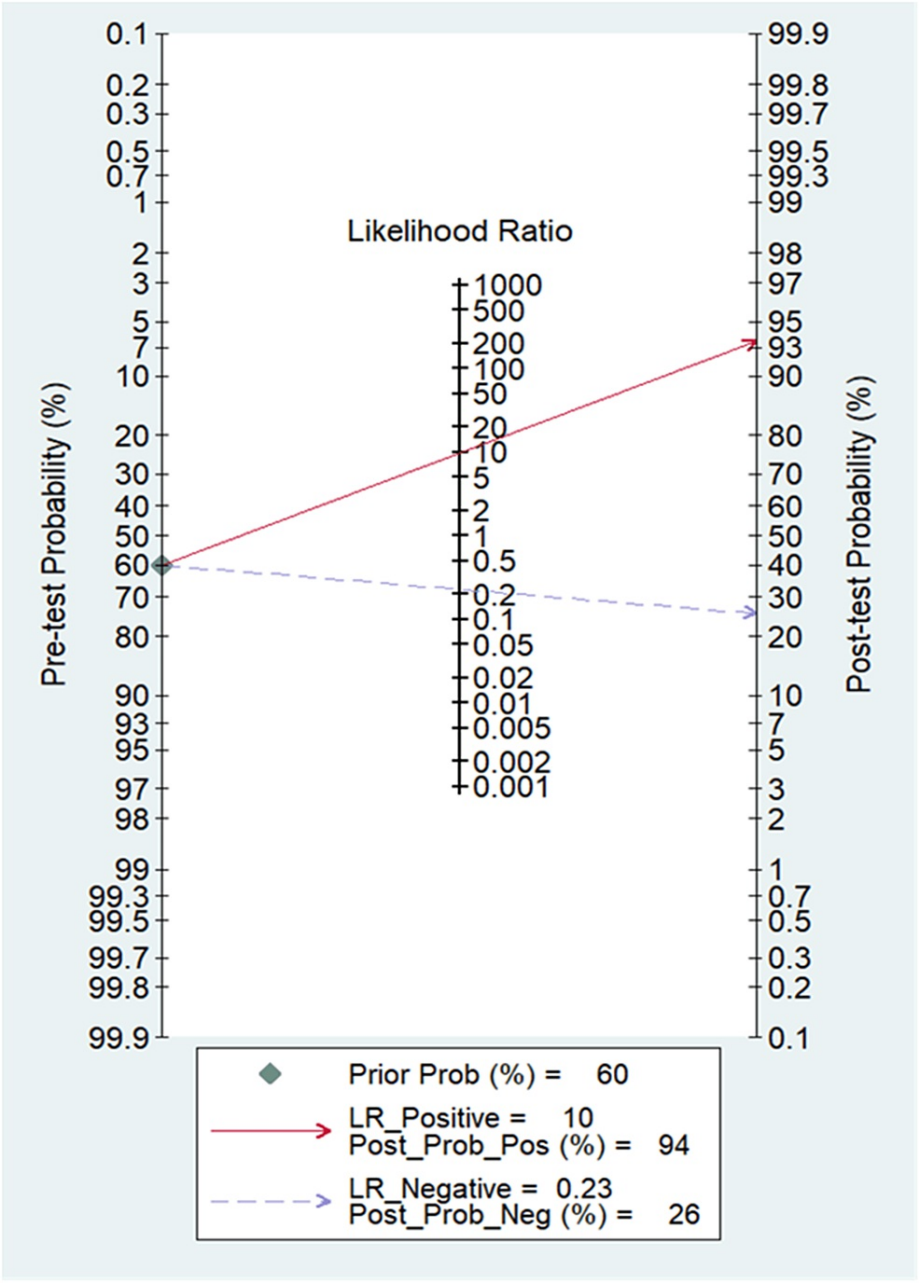
Fagan’s nomogram of serum HE4 Nomogram analysis shows the pre-test and post-test probability of serum HE4 in the diagnosis of ovarian cancer. HE4, human epididymis protein 4

**Figure 8:**
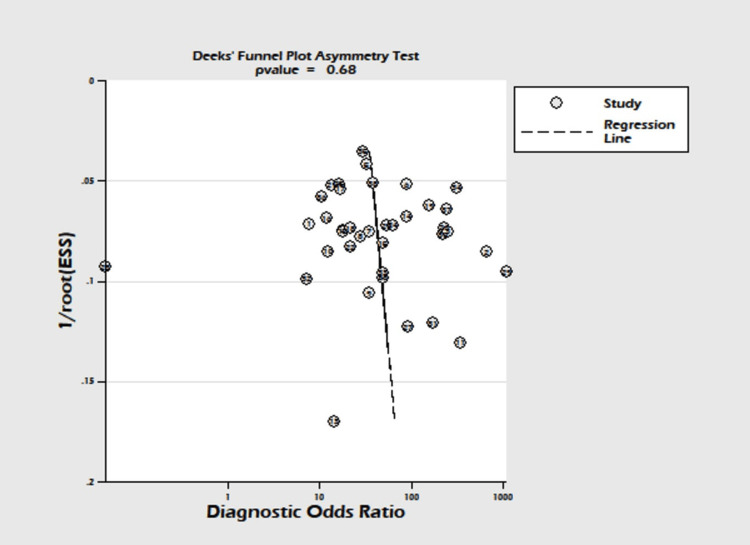
Deek’s funnel plot for assessing the risk of publication bias

A total of 38 studies involving 14,745 subjects were contributed for pooled analysis for determining the diagnostic accuracy of serum HE4 levels for ovarian cancer. Our analysis showed that serum HE4 levels had acceptable combined sensitivity (0.79; 95% CI: 0.75-0.82; I^2^=84%) and clinically meaningful specificity (0.92; 95% CI: 0.87-0.95; I^2^=98.7%) for differentiating ovarian cancer from the normal condition with reference to the gold standard as biopsy (Figure 5). The discriminating power of serum HE4 for ovarian cancer measured by the summary area under the curve (AUC) was promising (SROC: 0.88; 95% CI: 0.85-0.91) (Figure 6). In the case of a pre-test probability of 60% for disease positive, the post-test probability for serum HE4-positive subjects increased up to 94%, and on the other hand, the serum HE4-negative case may lead to post-test probability for positive result drops up to 26% (Figure 7). The diagnostic odds ratio for differentiating ovarian cancer from the normal subjects was 43 (95% CI: 23-72). The positive likelihood ratio was 9.7 (95% CI: 6-15.7), and the negative likelihood ratio of serum HE4 levels was 0.23 (95% CI: 0.19-0.27) for diagnosing ovarian cancer. We did not find evidence of significant publication bias (P=0.68) (Figure 8).

Result of Urine HE4 Meta-Analysis

**Figure 9:**
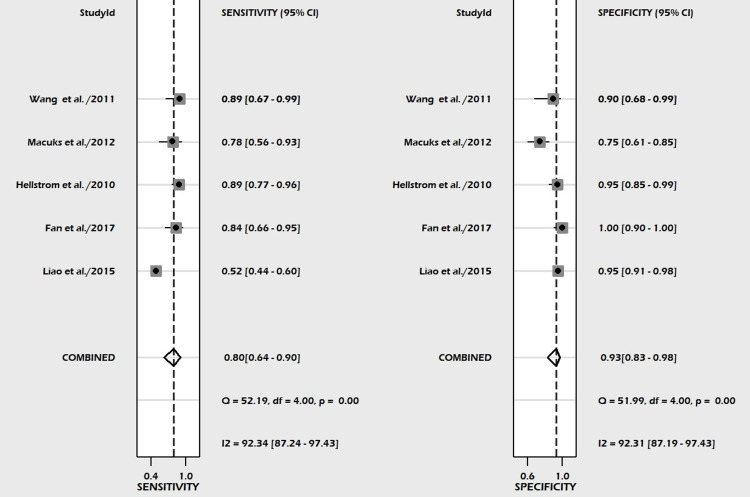
Forest plot of pooled sensitivity and specificity of urine HE4 in the diagnosis of ovarian cancer HE4, human epididymis protein 4; CI, confidence interval [57-61]

**Figure 10:**
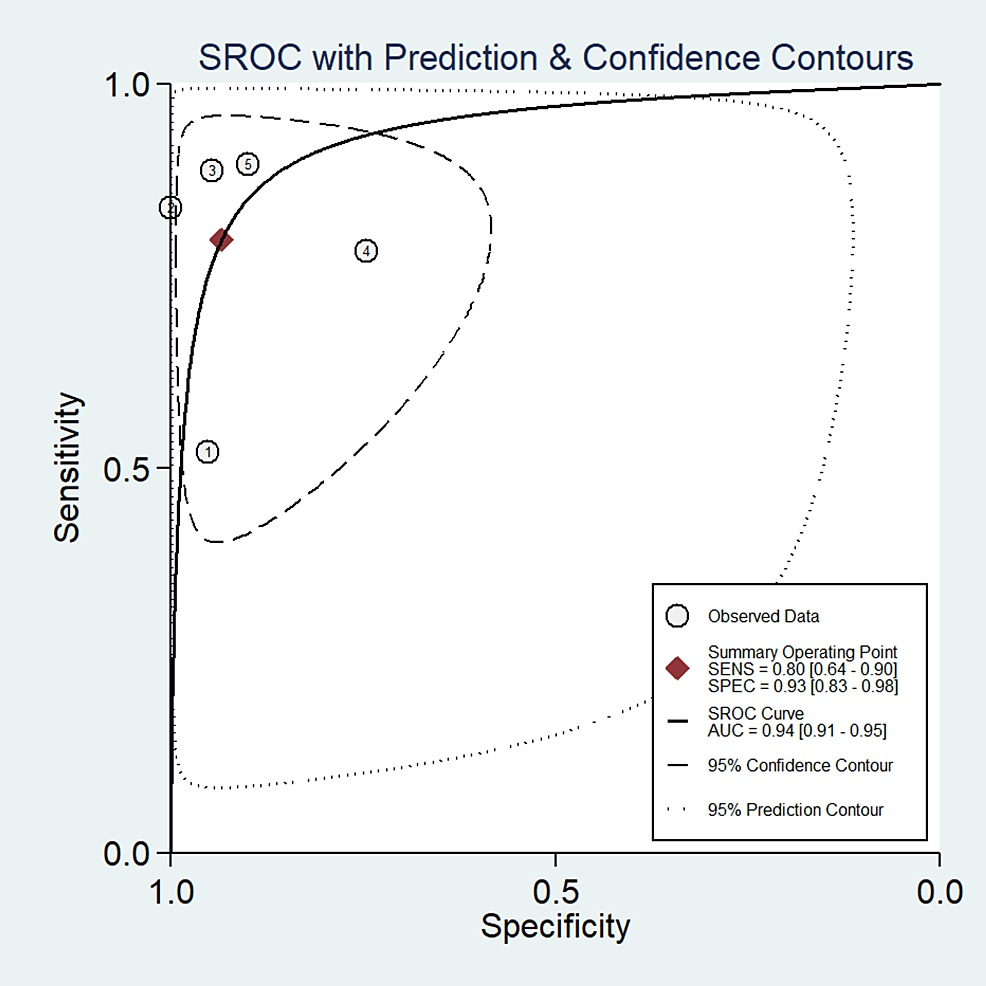
SROC curve with prediction and confidence contours showing the discriminatory power of urine HE4 for diagnosing ovarian cancer SROC, summary receiver operating characteristics; HE4, human epididymis protein 4; AUC, area under the curve

**Figure 11:**
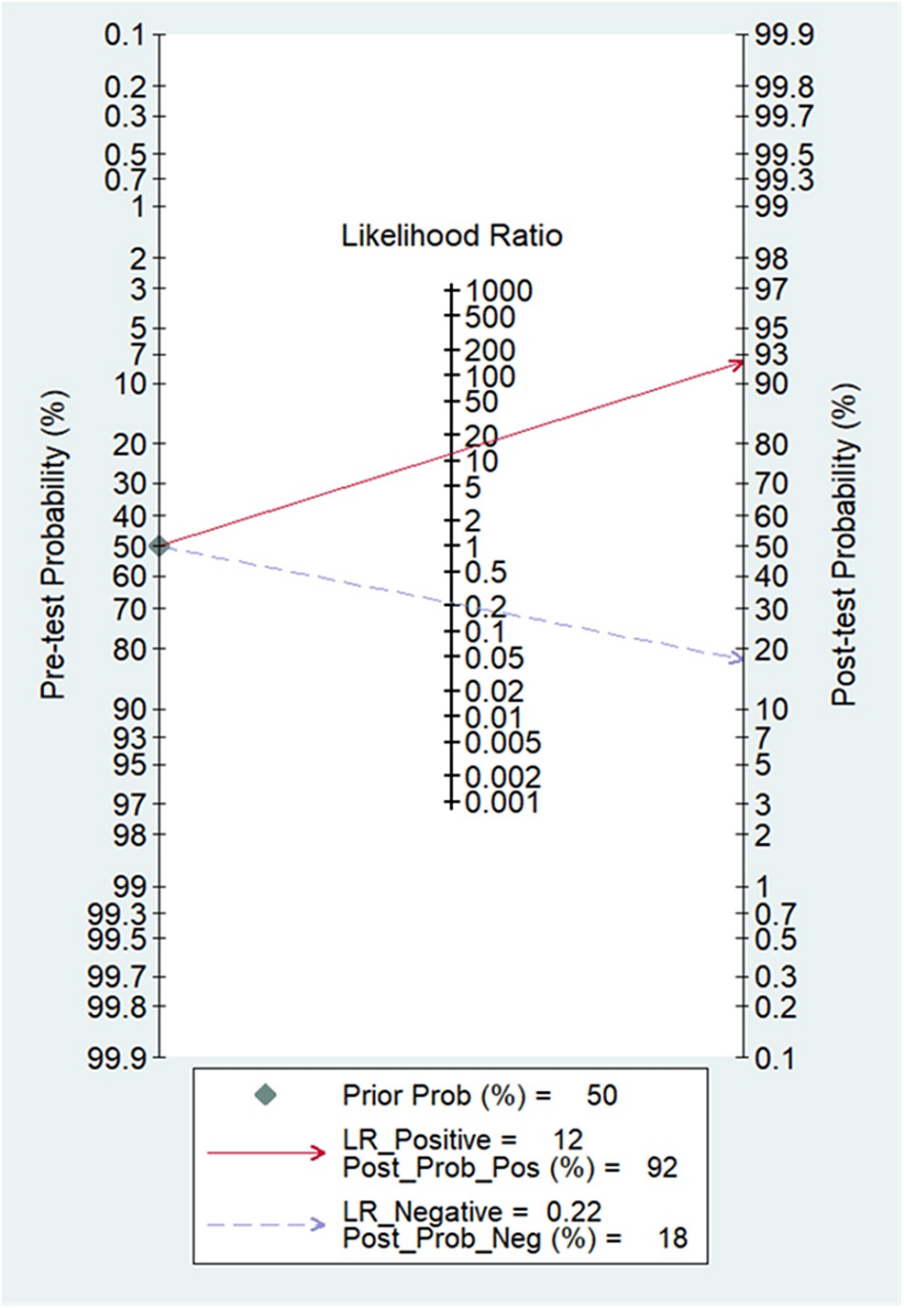
Fagan’s nomogram of urine HE4 Nomogram analysis shows the pre-test and post-test probability of urine HE4 in the diagnosis of ovarian cancer. HE4, human epididymis protein 4

A total of five studies involving 649 subjects were contributed for pooled analysis for determining the diagnostic accuracy of urine HE4 levels for ovarian cancer. Our analysis showed that urine HE4 levels had acceptable pooled sensitivity (0.80; 95% CI: 0.64-0.90; I^2^=92.3%) and higher specificity (0.93; 95% CI: 0.83-0.98; I^2^=92.3%) for differentiating ovarian cancer from the normal condition with reference to the gold standard as biopsy (Figure 9). The discriminating power of HE4 for ovarian cancer measured by SROC was 0.94 (95% CI: 0.91-0.95) (Figure 10). In the case of a pre-test probability of 50% for disease positive, the post-test probability for HE4-positive subjects increased up to 92%, and on the other hand, the HE4-negative case may lead to post-test probability for positive results decreased up to 18% (Figure 11). The DOR for differentiating ovarian cancer from the normal subjects was 55 (95% CI: 15-198). The positive likelihood ratio was 12 (95% CI: 4.5-31.6), and the negative likelihood ratio of urine HE4 levels was 0.22 (95% CI: 0.12-0.41) for diagnosing ovarian cancer. We could not examine the publication bias due to an insufficient number of studies on urine HE4 levels.

Meta-Regression and Subgroup Analyses for Serum HE4

**Figure 12:**
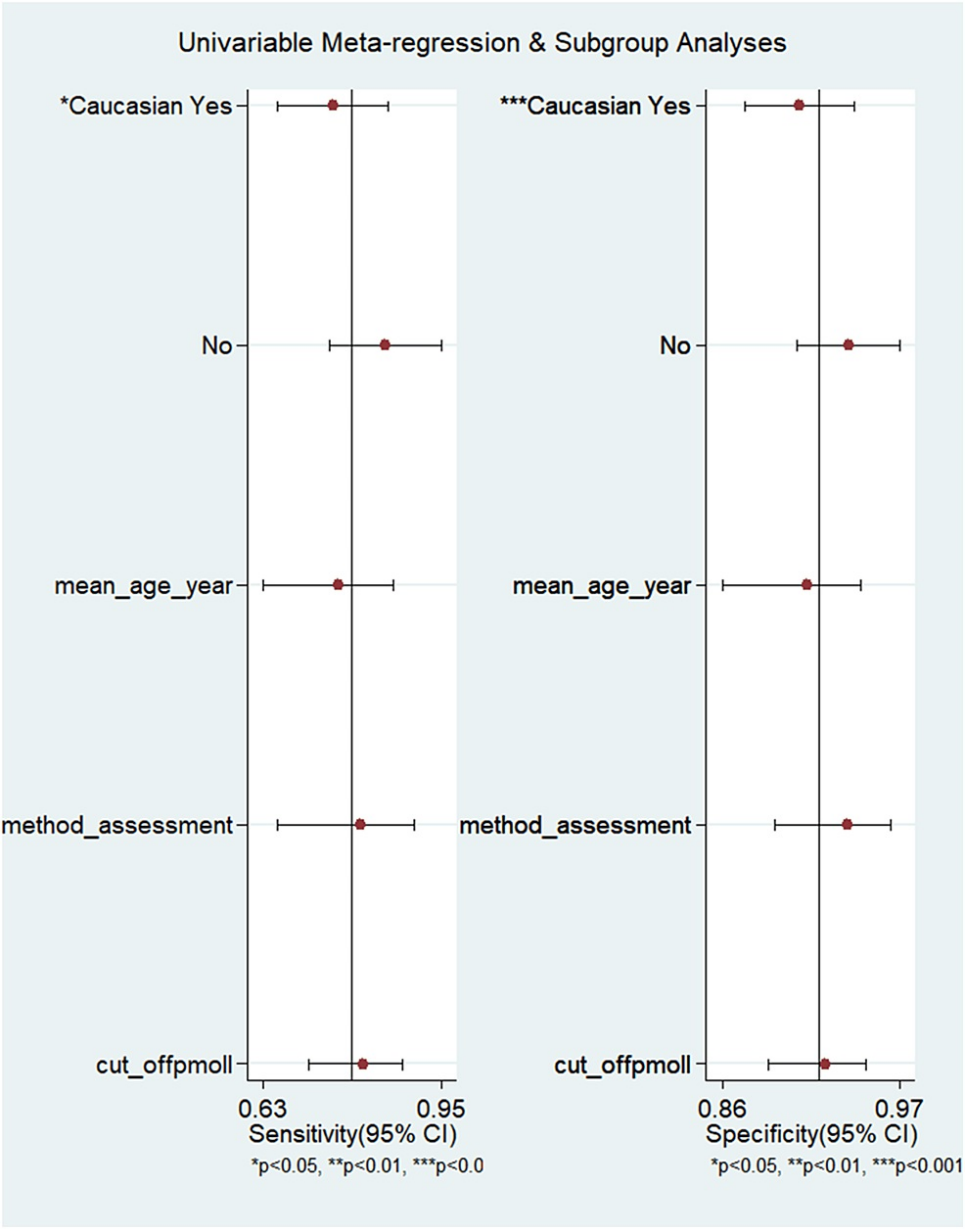
Meta-regression analysis (serum HE4) to find out the reason for heterogeneity HE4, human epididymis protein 4; CI, confidence interval

A crucial step in a meta-analysis is to assess the heterogeneity. Because the statistical model can be affected by the presence or absence of true heterogeneity, we carried out the meta-regression analysis to analyze the sources of heterogeneity and further exploration. Our analysis could not find a significant source of heterogeneity by the mean age, cutoff values, and methods of assessment (Figure 12). However, the heterogeneity was explained by ethnicity.

Discussion

Our updated meta-analysis examined the precise role of serum and urine HE4 as a diagnostic biomarker for ovarian cancer. Early diagnosis of ovarian cancer is very difficult because of its insidious onset and vague symptom. Approximately 70% of ovarian cancer is diagnosed in an advanced stage. The five-year survival rate of ovarian cancer depends upon the stage of diagnosis. For the last 30 years, CA-125 has been used for the diagnosis and monitoring of the recurrence of ovarian cancer. However, its low specificity as a tumor marker is the main disadvantage. CA-125 levels are increased in many benign conditions, including endometriosis, and also during pregnancy and menstrual cycle. Therefore, the sensitivity and specificity of CA-125 are further questionable in premenopausal women. This limits the utility of CA-125 as a tumor marker in premenopausal women. This is the reason HE4 has attracted much attention as a tumor marker. HE4 is an 11-kDa precursor protein. It is a precursor to the epididymal secretory protein 4, which is overexpressed in ovarian cancer patients. However, there is minimal gene expression and thus less production of HE 4 in normal ovarian tissue [4,62,63].

The currently available evidence of HE4, both serum and urine, as a tumor marker for the diagnosis and differentiation of ovarian masses is summarized in our meta-analysis. Figure 5 and Figure 9 show the forest plot of pooled sensitivity and pooled specificity of serum and urine HE4, respectively, for the diagnosis of ovarian cancer in our meta-analysis, which endorses the diagnostic efficacy of serum and urine HE4.

A previous meta-analysis by Zhen et al. [4], which included studies between 2008 and 2012, on the comparative role of CA-125 and HE4 as a biomarker in ovarian cancer revealed that the pooled sensitivity (95% CI) for HE4 and CA-125 were 0.74 (0.72-0.76) and 0.73 (0.72-0.76), respectively, which is lower than our updated meta-analysis. The pooled specificity of this meta-analysis (95% CI) for HE 4 and CA-125 were 0.87 (0.89-0.91) and 0.84 (0.81-0.84), respectively, which is also lower than our updated meta-analysis result of specificity of serum HE4.

The first promising efficacy of HE4 was documented by Yu et al. [8] in their meta-analysis in 2012. It was the first meta-analysis to compare the diagnostic efficacy of HE4 to CA-125. This meta-analysis concluded that HE4 was a better tumor marker than CA-125 for sensitivity, specificity, and LR+ and LR- values.

The result of DOR (serum HE4: 43 (95% CI: 23-72); urine HE4: 55 (95% CI: 15-198)) in our meta-analysis further reinforces the ability of HE4 to differentiate between benign and malignant ovarian tumors.

The value of SROC was also promising (0.88 (95% CI: 0.85-0.91) for serum HE4 (Figure 6) and 0.94 (95% CI: 0.92-0.95) for urine HE4 (Figure 10)). In 2014, another meta-analysis report was published by Macedo et al. [9] with endorsing results in favor of HE4 (AUC: 0.91) that this tumor marker is a useful predictor of benign and malignant ovarian tumors.

The likelihood ratio is also a measure of the reliability of the diagnostic test. In our meta-analysis, the value of pooled LR-positive for serum HE4 revealed that it was 9.7 (95% CI: 6-15.7), and for urine HE4, it was 12 (95% CI: 4.5-31.6), which is higher than the recommended level (5). The pooled LR-negative value for serum HE4 was 0.23 (95% CI: 0.19-0.27), and for urine HE4, it was 0.22 (95% CI: 0.12-0.41). LR values are shown in Figure 7 and Figure 11 for serum HE4 and urine HE4, respectively. LR-negative value implies the ability of HE4 for ruling out the disease.

There are different methods to assay HE4 levels, such as ELISA, a chemiluminescence microparticle immunoassay, and an electrochemiluminescence assay. ELISA is not only laboratory-intensive and time-consuming but also has lower analytical sensitivity and precision performance than automated assays. Also, the result of ELISA can be affected by the proficiency of the performer. In 2010, the FDA approved an automated HE4 assay to improve the drawback of ELISA. Therefore, not only an age-dependent cutoff value but a uniform, accurate method to assay HE4 is also required [64-66].

In the HPE of the included studies, all ovarian malignancies were serous epithelial type, except for one, which was a mixed variety. Similar was the result with urinary HE4 studies (all were serous epithelial, and one was of mixed variety). Suri et al. [64] in their meta-analysis also concluded that HE4 is a promising predictor of epithelial ovarian cancer in premenopausal women. A DOR of 41.03 (27.96-60.21), specificity of 0.90 (95% CI: 0.89-0.91), and area under the curve (AUC) of 0.91 for serum HE4 were the highest in the premenopausal women in her meta-analysis in comparison to the Risk Of Malignancy Algorithm (ROMA) and CA-125. However, Suri et al. found the ROMA as the best marker to differentiate epithelial ovarian cancer from a benign ovarian tumor in postmenopausal women, and their meta-analysis results for the ROMA were DOR of 44.04, sensitivity of 0.88, and AUC of 0.94, whereas the results for HE4 were DOR of 41.03, sensitivity of 0.73, and AUC of 0.91. The probable explanation is that the ROMA, a qualitative algorithm, considers serum CA-125, serum HE4, and the menopausal status of women to calculate its value. However, serum CA-125 is not a reliable tumor marker in premenopausal women [4,5,33,66].

Strength of the Study

To make the study reliable and robust, we stuck to the guidelines for meta-analysis of diagnostic accuracy of both serum and urine HE4. This is the main strength of the study. We included many recent studies also. We followed the PRISMA guidelines for the selection of studies. Strict methodological quality assessments of all studies were done according to QUADAS-2 tools. Meta-regression and subgroup analyses were performed to explore a potential source of heterogeneity.

Limitations of the Study

The present study only included studies in English literature only, which could allow language bias in study selection. Many included studies used ELISA as a method of assay for HE4, which have its own limitations. Therefore, the inclusion of studies in which the automated method was used might be better for the reliability and uniformity of results. We could include only five studies (after exclusion) for urine HE4. Hence, more studies on the role of urine HE4 are required to reach a solid conclusion. In our included studies, there was no stratification of the HE4 level according to the stage of the malignancy and histopathological type of ovarian tumor. Therefore, this was also a limitation of our meta-analysis due to insufficient studies. The HE4 level also increases in smokers and females who are on oral contraceptives. However, in the included studies, it was not mentioned in the history/clinical profile of the subjects.

## Conclusions

The current meta-analysis concluded that serum HE4 is a promising biomarker with a high degree of specificity and acceptable sensitivity for the diagnosis of ovarian cancer (especially epithelial ovarian cancer). However, more studies are required for international standardization and to establish an age group-wise cutoff level of HE4, a separate cutoff level for premenopausal and postmenopausal women, and an ethnicity-based cutoff level for HE4. Further studies and explorations are also required to establish a single method to assay HE4 so that a uniform and standard interpretation of the result will be possible. More studies are also required to establish the diagnostic role of urinary HE4 because if we get a promising, convincing result, it will be a more compliant diagnostic test for patients. Thus, we are concluding with the hope that in the future we will have an ideal/near-ideal diagnostic test to screen and diagnose women with early ovarian cancer and thus reduce mortality.
